# The index lift in data mining has a close relationship with the association measure relative risk in epidemiological studies

**DOI:** 10.1186/s12911-019-0838-4

**Published:** 2019-06-17

**Authors:** Khanh Vu, Rebecca A. Clark, Colin Bellinger, Graham Erickson, Alvaro Osornio-Vargas, Osmar R. Zaïane, Yan Yuan

**Affiliations:** 1 University of Alberta School of Public Health, Edmonton, AB Canada; 2 Department of Computing Science, University of Alberta, Edmonton, AB Canada; 3 University of Alberta Women and Children’s Health Research Institute, Edmonton, AB Canada; 4 Department of Pediatrics, University of Alberta, Edmonton, AB Canada

**Keywords:** Lift, Relative risk, Odds ratio, Data mining, Association rule mining, Interestingness measures, Air pollution, Environmental health

## Abstract

**Background:**

Data mining tools have been increasingly used in health research, with the promise of accelerating discoveries. Lift is a standard association metric in the data mining community. However, health researchers struggle with the interpretation of lift. As a result, dissemination of data mining results can be met with hesitation. The relative risk and odds ratio are standard association measures in the health domain, due to their straightforward interpretation and comparability across populations. We aimed to investigate the lift-relative risk and the lift-odds ratio relationships, and provide tools to convert lift to the relative risk and odds ratio.

**Methods:**

We derived equations linking lift-relative risk and lift-odds ratio. We discussed how lift, relative risk, and odds ratio behave numerically with varying association strengths and exposure prevalence levels. The lift-relative risk relationship was further illustrated using a high-dimensional dataset which examines the association of exposure to airborne pollutants and adverse birth outcomes. We conducted spatial association rule mining using the Kingfisher algorithm, which identified association rules using its built-in lift metric. We directly estimated relative risks and odds ratios from 2 by 2 tables for each identified rule. These values were compared to the corresponding lift values, and relative risks and odds ratios were computed using the derived equations.

**Results:**

As the exposure-outcome association strengthens, the odds ratio and relative risk move away from 1 faster numerically than lift, i.e. |log (odds ratio)| ≥ |log (relative risk)| ≥ |log (lift)|. In addition, lift is bounded by the smaller of the inverse probability of outcome or exposure, i.e. lift≤ min (1/P(O), 1/P(E)). Unlike the relative risk and odds ratio, lift depends on the exposure prevalence for fixed outcomes. For example, when an exposure A and a less prevalent exposure B have the same relative risk for an outcome, exposure A has a lower lift than B.

**Conclusions:**

Lift, relative risk, and odds ratio are positively correlated and share the same null value. However, lift depends on the exposure prevalence, and thus is not straightforward to interpret or to use to compare association strength. Tools are provided to obtain the relative risk and odds ratio from lift.

**Electronic supplementary material:**

The online version of this article (10.1186/s12911-019-0838-4) contains supplementary material, which is available to authorized users.

## Background

Readily available large administrative databases greatly facilitate the utilization of data mining algorithms in health research, promising the acceleration of knowledge discoveries [1, 2]. Data mining algorithms use indices of so-called “interestingness” to generate and select association rules from complex high dimensional datasets [3]. One widely used index is “lift” [4]. In the data mining literature, lift is the ratio of the joint occurrence of antecedent, X, and consequent, Y, to the product of marginal occurrences of X and Y, adjusting for the number of total records, i.e. $\frac{P(XY)}{P(X)P(Y)}$ [5]. Lift has been used to identify risk factors associated with acute myocardial infarction [6], rheumatoid arthritis [7], and cancer survival [8, 9], as well as to detect signals of adverse drug events [10].

The relatively new concept lift has created a barrier for interpretation of results by health researchers, which was discovered during the course of our interdisciplinary data mining project [11, 12]. Our multi-disciplinary team included computer scientists, statisticians, epidemiologists, neonatologists, and pediatricians. Team members coming from a health background were familiar with the classic epidemiological measures of association such as the relative risk and odds ratio. The health researchers expressed their desire to better understand what lift represents. Previously, lift and odds ratio have been discussed in the context of their appropriateness for ranking association rules and improving the efficiency of data mining processes [13–15]. However, there has been no discussion of the lift-relative risk or lift-odds ratio relationship that assists interpretation, especially for health researchers. Our objective in this paper is to derive equations that link lift with the relative risk and odds ratio. By establishing these relationships, we bridge the gap between data mining and health research. This work will facilitate the comprehension of lift by health researchers, and relative risk by computer scientists.

The remainder of the article is organized as follows. In the *Methods and Results*, we briefly visit the definitions of lift and relative risk in the context of their respective fields of research. Using a 2 by 2 contingency table, we first derive the equation connecting lift and relative risk. Based on the derived equation, we discuss the theoretical relationship between lift and relative risk when the association strength and exposure prevalence changes. Next, we illustrate the relationship empirically in the *Neonatal Birth Outcomes Example*. Lastly, the strengths and limitations of each measure are discussed in the *Discussion* and *Conclusions*.

## Methods and results

### Theoretical derivation

#### Definitions and notations

As mentioned previously, lift is the ratio of the joint occurrence of an antecedent, X, and a consequent, Y, to the product of the marginal occurrences of X and Y, adjusting for the number of total records, i.e. $\frac{P(XY)}{P(X)P(Y)}$ [5]. It evaluates the X-Y association: when X and Y are independent, lift is equal to 1. When X and Y are positively correlated, lift > 1. A negative correlation between X and Y implies lift < 1. A lift value further from 1 implies a stronger association between X and Y.

In the field of epidemiology, the relative risk is the ratio of the event (or consequent, Y) occurrence in subjects who are exposed to X (antecedent) and the event occurrence in the non-exposed subjects, adjusting for the total number of exposed and non-exposed subjects, i.e. $\frac{P\left(Y|X\right)}{P\left(Y|\overline{X}\right)}$. Here, X denotes exposure to the antecedent(s) and $\overline{X}$ denotes non-exposure to the antecedent(s).

It is convenient to illustrate these concepts using a 2 by 2 contingency table (Table 1). Following traditional epidemiological notation, we use the terms outcome (O) and exposure (E), analogous to consequent, Y, and antecedent, X, respectively.

**Table 1:** Contingency table based on counts

	Outcome (Yes)	Outcome (No)	Total
Exposure (Yes)	a	b	a + b
Exposure (No)	c	d	c + d
Total	a + c	b + d	N = a + b + c + d

In the data mining literature, the following indices are defined

$$ {lift}_{\left(O|E\right)}\overset{\mathrm{def}}{=}\frac{P\left(O|E\right)}{P(O)}\overset{\mathrm{def}}{=}\frac{P(OE)}{P(O)P(E)}=\frac{aN}{\left(a+c\right)\left(a+b\right)} $$

$$ support\overset{\mathrm{def}}{=}P(OE)=\frac{a}{N} $$

$$ confidence\overset{\mathrm{def}}{=}P\left(O|E\right)=\frac{a}{a+b} $$

Note that *lift*_(*E*| *O*)_ = *lift*_(*O*| *E*)_. We chose to use the notation *lift*_(*O*| *E*)_ in this article to stress the casual implication of outcome given exposure. To simplify notations, hereafter lift is used to refer to *lift*_(*O*| *E*)_. From the definition of lift, we note that lift $\le \mathit{\min}\left(\frac{N}{a+b},\frac{N}{a+c}\right)=\mathit{\min}\left(\frac{1}{P(E)},\frac{1}{P(O)}\right)$, because both b and c ≥ 0.

In epidemiology, the relative risk is defined as

$$ RR\overset{\mathrm{def}}{=}\frac{P\left(O|E\right)}{P\left(O\right|\overline{E}\Big)}=\frac{\frac{a}{a+b}}{\frac{c}{c+d}} $$

The relative risk and lift relationship can be expressed as

$$ RR=\frac{\left(1-P(E)\right) lift}{1-P(E) lift}\ (1) $$

The derivation of equation (1) is given in Additional file 1: Appendix 1, which shows that the relative risk can be obtained from *lift*_(*O*| *E*)_ and the proportion of exposed subjects, *P*(*E*).

Another commonly used association measure in epidemiology is the odds ratio. The lift-odds ratio relationship is

$$ OR\overset{\mathrm{def}}{=}\frac{P\left(O|E\right)/P\left(\overline{O}|E\right)}{P\left(O|\overline{E}\right)/P\left(\overline{O}|\overline{E}\right)}=\frac{a/b}{c/d}=\frac{\frac{\left(1-P(E)\right) lift}{1-P(E) lift}-P(O) lift}{1-P(O) lift}\ (2) $$

The derivation can be found in Additional file 1: Appendix 2. In the following discussion, we focus mainly on the lift-relative risk relationship because of three considerations. First, the characteristics of the lift-odds ratio relationship are similar to those of the lift-relative risk relationship. Second, when comparing the relative risk and odds ratio, the relative risk is the preferred measure of association strength as its interpretation is more straightforward [16, 17]. It is worth noting that in the case of rare outcomes, the odds ratio and relative risk are similar numerically [16, 17]. Third, the typical design of studies which use data mining tools allows for the calculation of both relative risk and odds ratio. Association rule mining is commonly used in high dimensional large administrative databases (e.g. electronic medical records or patient claims data) for a specific cohort or population. In these studies, the prevalence of the outcome is not fixed by design and has a meaningful interpretation. Therefore, both the relative risk and odds ratio are allowable measures in data mining studies, unlike in epidemiological case-control studies where only the odds ratio can be estimated.

#### The lift – relative risk relationship

The similarity between lift and the relative risk is apparent when equation (1) is rearranged

$$ RR-1=\frac{lift-1}{1-P(E) lift}\ (3) $$

When lift equals 1, the relative risk is also equal to 1, implying no association between exposure and outcome. As the denominator 1 − *P*(*E*)*lift* always takes values between 0 and 1 (proof in Additional file 1: Appendix 3), lift and relative risk are greater than 1 simultaneously when the exposure positively correlates with the outcome. When the exposure negatively correlates with the outcome, both lift and relative risk are less than 1. Since the odds ratio and the relative risk change in unison, lift, relative risk, and odds ratio have the same null value of 1, and change in the same direction with respect to positive and negative correlation between outcome and exposure.

**Fig. 1:**
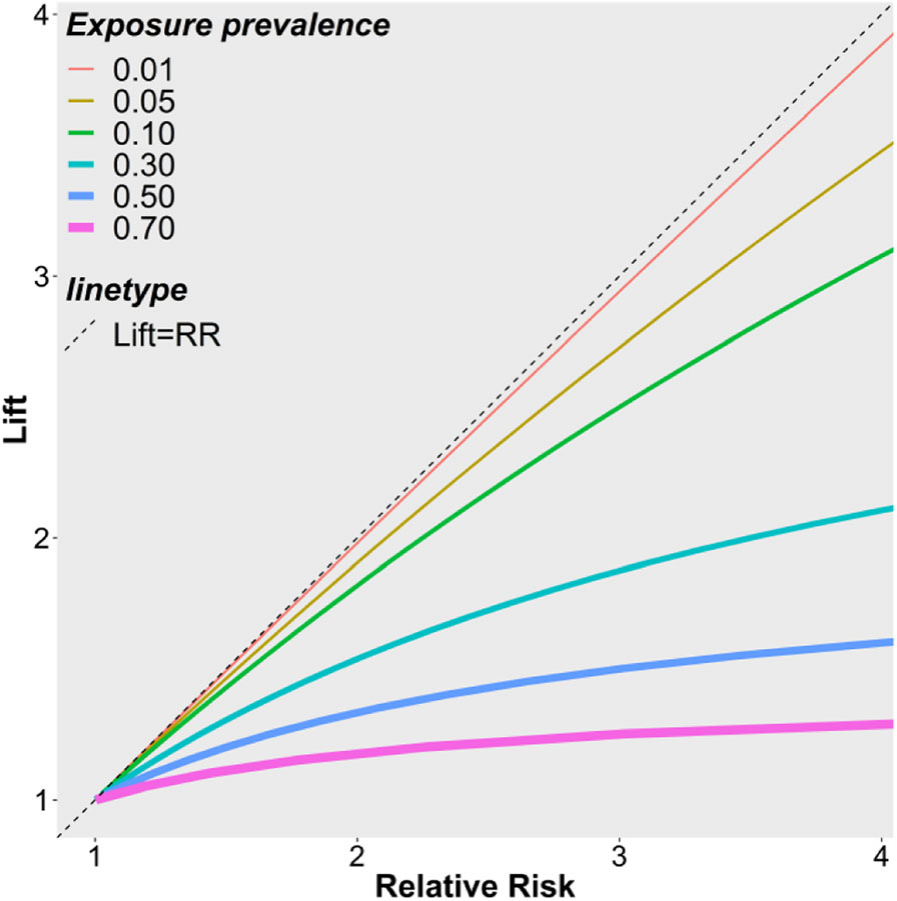
Relationship between relative risk and lift. For each exposure prevalence value, lift ≤1/P(E). Therefore, for P(E) = 0.01, 0.05, 0.1, 0.3, 0.5 and 0.7, the upper limits of lift are less than or equal to 100, 20, 10, 3.3, 2 and 1.4, respectively

The relative risk is always further from the null value of 1 than lift in both directions when an association exists between outcome and exposure. The relative risk is greater than lift for positively correlated outcomes and exposures, and smaller than lift for negatively correlated outcomes and exposures (Additional file 1: Appendix 3). As the odds ratio is farther from the null than the relative risk when an association exists [18], |log (odds ratio)| ≥ |log (relative risk)| ≥ |log (lift)| holds. Furthermore, the ratio of relative risk/lift is close to 1 when the exposure is rare, i.e. *P*(*E*) is low (Additional file 1: Appendix 3). The relationship between the relative risk and lift for varying exposure prevalence levels and association strengths are shown in Fig. 1. Concave lines suggest that as the strength of the positive association increases, the ratio of relative risk/lift increases (proof in Additional file 1: Appendix 4). The ratio of relative risk/lift also increases with increasing exposure prevalence (proof in Additional file 1: Appendix 5 and supporting theoretical data in Additional file 1: Appendix 6).

#### From lift to relative risk – implementing the conversion

To obtain relative risk, equation (1) can be easily implemented in popular software packages, such as Microsoft Excel, Stata, SAS, or R. We provide a tool that converts the data mining indices lift, support, and confidence from the output of existing data mining packages to the relative risk and odds ratio (Additional file 1: Appendix 7). An R function and SAS macro are included in Additional file 1: Appendix 8 and Additional file 1: Appendix 9. These functions are also available on our website, https://sites.ualberta.ca/~yyuan/software.html. Conveniently, the data mining package <arules> in R, which implements the Apriori algorithm, outputs the odds ratios with the “interestMeasure” function along with the usual data mining indices [19].

### Neonatal birth outcome example

**Fig. 2:**
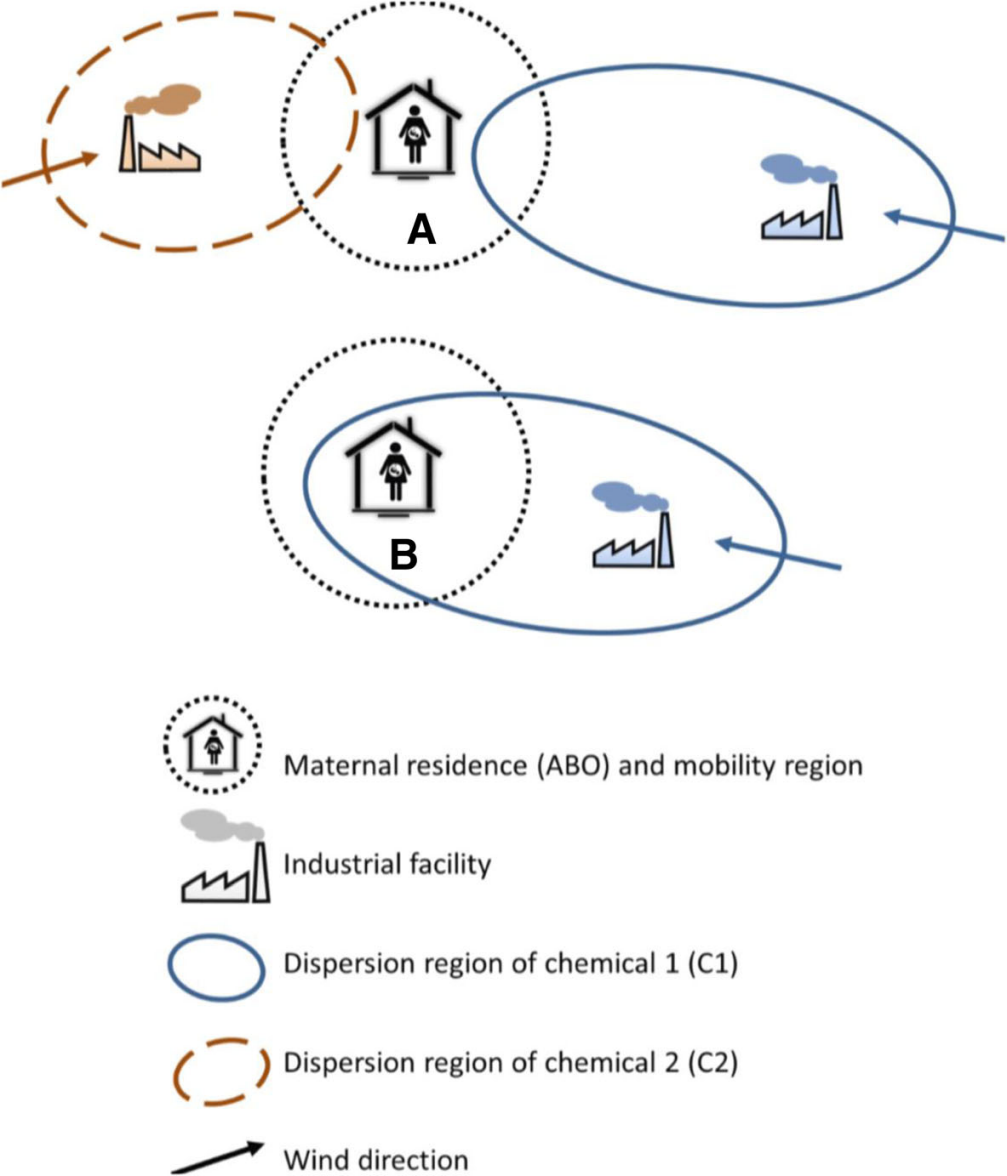
Illustration of spatial data mining algorithm assigning airborne chemicals exposure to births. The assignment of airborne chemical exposure to the births was based on the maternal residences, chemical emission sources and wind information. In this illustration, subject A is exposed to both C1 and C2. Subject B is exposed to C1

We conducted association rule mining using a real world dataset to illustrate the connection between lift and the relative risk. Values for lift and the relative risk were calculated directly for selected rules from the raw data. They were cross-tabled (Table 3) and plotted (Fig. 2). Directly calculated relative risks were also compared to relative risks converted from lift using equation (1). These results empirically verified the lift-relative risk relationship as discussed above.

#### The DoMiNO dataset

The dataset used in this example is from the Data Mining & Neonatal Outcomes (DoMiNO) study. The DoMiNO study uses spatial association rule mining to identify mixtures of industrial airborne chemicals associated with adverse birth outcomes (ABO). The ABOs of interest are small for gestational age (SGA), low birth weight at term (LBWT), and preterm birth (PB) [11, 12].

The birth data was obtained from the population based Alberta Perinatal Health Program (APHP) in the Canadian province of Alberta [20]. In addition to all hospital births, APHP collects birth information from planned home births, and unplanned deliveries outside a facility [20]. We included 333,250 singleton live births from 2006 to 2012. To obtain prenatal exposure to industrial airborne chemicals during the same period, we used industrial emissions data reported by 6279 Alberta facilities to the National Pollutant Release Inventory (NPRI) [21], and wind pattern data from 182 stations in Alberta Agriculture’s AgroClimatic Information System 2010 [22].

Of the 333,250 total births, there were 29,679 SGA births, 22,733 LBWT births, and 5485 PB births recorded. As for airborne pollutants, a total of 136 chemicals from the industry activities were registered in NPRI during the study period. The location of the emission sites, the average emission amount, and the predominant wind (direction and speed) at each site were used to create a dispersion region for the chemicals [23]. A pregnant woman was considered exposed to a chemical if her activity area (a 5 km radius from the center of the postal code of her residence) overlapped with the dispersion region of the chemical (Fig. 2). Each birth (with ABOs) and exposure to chemicals served as a “transaction” for the association rule mining. The association rules to be mined by the data mining algorithm took the form “chemical(s) ➔birth outcome”.

#### Lift and relative risk of the mined association rules

Association rules between exposure to combinations of up to 8 chemicals and each type of ABO were mined using the Kingfisher algorithm [24, 25]. The algorithm uses Fisher’s exact test and a statistical significance level of 0.05 to identify positive association rules, i.e. lift > 1. The algorithm identified a total of 10,788 significant rules, with a range of lift from 1.00 to 1.53 and a range of exposure prevalence from 0.08 to 98.73%. Relative risks and odds ratios of these identified rules were directly calculated by cross-tabulation of the raw DoMiNO data for the corresponding exposures and outcomes. For example, one identified rule is an SGA birth and exposure to a mixture of carbon disulphide, carbonyl sulphide, and toluene (Table 2). The exposed group consisted of pregnant women exposed to all three chemicals, and the non-exposed group consisted of pregnant women that either had no exposure to any of the three chemicals, or were exposed to only one or two of the three chemicals.

**Table 2:** An example

Exposure	Outcome		Total
	SGA Birth	Non-SGA Birth	
Mixture of carbon disulphide, carbonyl sulphide, and toluene (exposure group)	7828	64,021	71,849
None, or any one or two of these three chemicals (non-exposure group)	21,851	239,550	261,401
Total	29,679	303,571	333,250

For the example in Table 2, the exposure and outcome prevalence, support, confidence, lift, relative risk, and odds ratio for the mixture of the three chemicals are

$$ P(E)=\frac{\mathrm{71,849}}{\mathrm{333,250}}=0.22 $$

$$ P(O)=\frac{\mathrm{29,679}}{\mathrm{333,250}}=0.089 $$

$$ support=\frac{\mathrm{7,828}}{\mathrm{333,250}}=0.023 $$

$$ confidence=\frac{\mathrm{7,828}}{\mathrm{71,849}}=0.11 $$

$$ lift=\frac{\mathrm{7,828}}{\mathrm{71,849}}/\frac{\mathrm{29,679}}{\mathrm{333,250}}=1.22 $$

$$ RR=\frac{\mathrm{7,828}}{\mathrm{71,849}}/\frac{\mathrm{21,851}}{\mathrm{261,401}}=1.30 $$

$$ OR=\frac{\mathrm{7,828}}{\mathrm{64,021}}/\frac{\mathrm{21,851}}{\mathrm{239,550}}=1.34 $$

We note that the odds ratio estimate from this 2 by 2 table was equal to the odds ratio estimate from an unadjusted logistic regression, treating pregnant women exposed to none, or any one or two of these three chemicals as the non-exposure group. In this example, the exposure group was composed of 22% of pregnant women who were exposed to all three chemicals (carbon disulphide, carbonyl sulphide, and toluene), i.e. P(E) = 0.22. Of all pregnant women in the study population, 8.9% had SGA births (P(O) = 0.089). Among the exposure group, 11% had SGA births (confidence = 0.11). The probability of having SGA births among the exposure group was 22% higher than the probability of having SGA births in the study population (lift = 1.22). The relative risk and odds ratio of having SGA births comparing the exposure to non-exposure groups were 1.30 and 1.34, respectively. This example shows that when there is a positive association between the exposure and outcome, odds ratio > relative risk > lift > 1.

In Table 3, the numerical relationship of lift-relative risk is expressed with the ratio $\frac{relative\ risk}{lift}$, stratified by exposure prevalence and lift values. All ratios are greater than or equal to 1.00. Relative risk/lift ratios range from 1.00 to 1.19.

**Table 3:** The ratio of relative risk versus lift, stratified by prevalence of the exposure and the lift value for rules identified by the Kingfisher algorithm in the DoMiNO study

P(E)	Lift					
	1.05	1.10	1.20	1.30	1.40	1.50
0.05	1.00	1.01	1.01	1.01	1.02	1.04
0.10	1.00	1.01	1.02	1.04	1.06	1.06
0.15	1.01	1.02	1.03	1.06	1.07	65
0.20		1.02	1.06	1.07		
0.25		1.04	1.07			
0.30	1.02	1.05	1.10			
0.35	1.03	1.06	1.11			
0.40	1.03	1.06	1.12			
0.45	1.04	1.10	1.14			
0.50	1.06	1.12				
0.55	1.06	1.15				
0.60	1.08	1.17				
0.65	1.08	1.18				
0.70		1.19				N/A

Blank cells mean no rule exists that satisfies the combination of lift and exposure. N/A means the combination of lift value and exposure prevalence is impossible

**Fig. 3:**
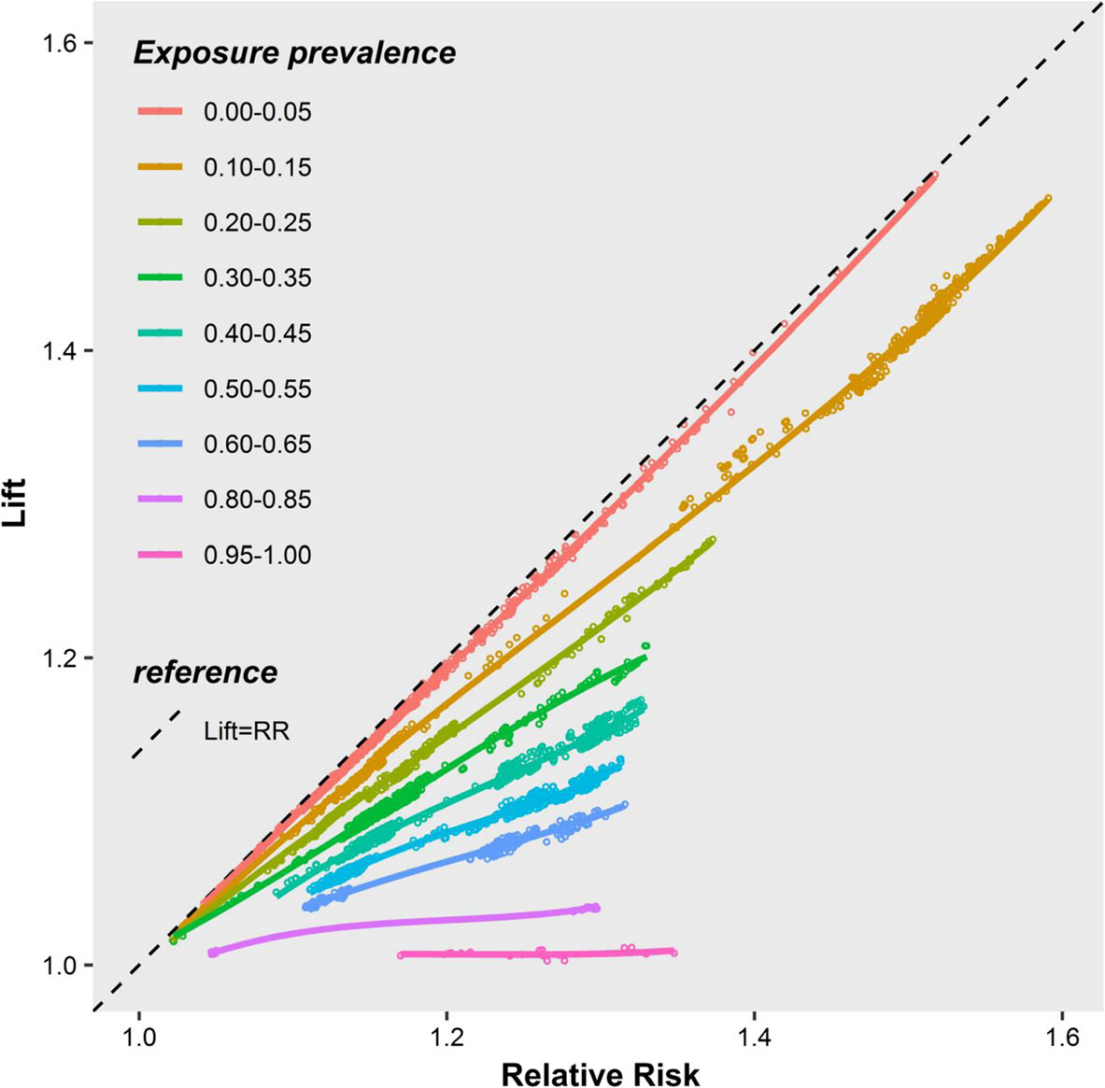
Relative risks and lifts obtained from the DoMiNO study using the Kingfisher algorithm. Each point plots a pair of lift and relative risk value corresponding to one rule. A total of 6282 of the 10,788 rules were included. Each line is the LOESS line for all points within each exposure prevalence group. The median exposure prevalence in each group was 0.04, 0.12, 0.22, 0.33, 0.43, 0.53, 0.62, 0.84, and 0.96

The exposure prevalence of the 10,788 rules varied widely. To visualize the empirical lift-relative risk relationship using the DoMiNO data, the exposure prevalence was divided into narrow intervals to group the rules. These narrow intervals ensured that the exposure prevalence within each group of rules was similar. A different color was assigned to each group. For each rule, the corresponding lift and relative risk values were plotted as one data point with its group color scheme in Fig. 3. After the scatter plot was generated, a LOESS line was fit and overlaid for each group.

Figure 3 is very similar to Fig. 1: 1) all lines are below the diagonal corresponding to lift = relative risk; and 2) the higher the exposure prevalence, the further the corresponding line is from the diagonal line. The lines in Fig. 3 look linear rather than concave simply because the range of the relative risk (1 to 1.6) is limited.

Relative risks and odds ratios for each association rule were also computed using equations (1, 2), using the lift, confidence, and support values output by the Kingfisher algorithm. The relative risks obtained using equation (1) were numerically indistinguishable from the relative risks obtained directly from the cross-tabulation of the raw DoMiNO data. This was also true for the odds ratios obtained using the two different approaches.

## Discussion

The standard measures of association in the health domain are the relative risk and odds ratio. A measure of association in data mining, lift, has recently been used in health research as interdisciplinary investigations become more common. In this article, we derived equations connecting the data mining indices lift, confidence, and support to the epidemiological association measures relative risk and odds ratio. The relationship between lift and relative risk was examined and demonstrated, both theoretically and empirically. We also implemented these equations in software packages widely used by health researchers. R and SAS functions were provided that convert indices directly from the output of data mining packages to relative risk and odds ratio values for easy interpretation by health researchers.

The relative risk and odds ratio have important advantages as measures of association strength in health research. These measures compare the likelihood of outcome occurrence between exposed and non-exposed groups. As a result of this formularization, the relative risk and odds ratio do not depend on the exposure prevalence [26]. Conceptually, the relative risk and odds ratio align well with the epidemiological causal framework based on the counterfactual theory [27]. As both measures are independent of the exposure prevalence, relative risk and odds ratio are comparable across studies and populations. These features make the interpretation and comparison of relative risks and odds ratios straightforward. Statistical models have been developed to estimate relative risk and odds ratio values associated with an exposure adjusting for confounders. The ability to isolate the effect of individual exposures is critical in health research under the causal framework.

Lift compares the likelihood of outcome occurrence in an exposed group with the likelihood of outcome occurrence in the entire study population. This makes its calculation straightforward and computationally efficient, especially when assessing the combined effect of multiple exposures.

However, equation (1) and Fig. 1 demonstrated that lift depends on the exposure prevalence. This has important implications for ranking rules based on lift and interpreting the association strength measured by lift. Suppose two exposures A and B have the same relative risk for preterm birth. The lift for the more prevalent exposure A will be lower than the lift for the less prevalent exposure B. Thus, exposure A is less “appreciated” by lift-based ranking algorithms, which can be misleading. From a public health perspective, the more prevalent exposure A will lead to a larger health burden due to preterm birth, and should be prioritized for intervention over exposure B. As a result, lift-based ranking algorithms may discard important rules that are high in both relative risk and exposure prevalence. Mining algorithms for health studies should consider accounting for exposure prevalence when ranking potential rules, e.g. converting lift to relative risk and using the relative risk to rank. Doing so borrows strength across disciplines and enhances the power of data mining for health research.

## Conclusions

Data mining is typically part of an exploratory data analysis, which is performed to generate scientific hypotheses. We should take advantage of the computational efficiency of lift and the ability of data mining tools to process large amounts of data. We suggest converting lift to the relative risk during the process of mining health data when the objective is to screen for “interesting” exposures. Ranking exposures according to their relative risks will make the ranking robust to exposure prevalence, and improve the interpretability of the identified associations. Due to the hypothesis generating nature of data mining, identified associations should be investigated in follow-up confirmatory multivariable regression analyses, and validated with external data.

## Additional file

Additional file 1: Appendix 1: Relative risk derivation. Appendix 2: Odds ratio derivation. Appendix 3: Relative risk versus lift. Appendix 4: Trend of relative risk-lift ratio by association strength. Appendix 5: Trend of relative risk-lift ratio by exposure prevalence. Appendix 6: Theoretical relationship of lift-relative risk and lift-odds ratio for selected lift and exposure prevalence combinations. Appendix 7: Obtaining relative risk and odds ratio from the output of the Kingfisher and R arules packages. Appendix 8: R code for obtaining relative risk and odds ratio from lift, support, and confidence. Appendix 9: SAS code for obtaining relative risk and odds ratio from lift, support and confidence. (DOCX 63 kb)

## Data Availability

The data that support the findings of this study are available from the University of Alberta but restrictions apply to the availability of these data, which were used under license for the current study, and so are not publicly available.

## Abbreviations

ABO: Adverse birth outcome
DoMiNO: Data Mining & Neonatal Outcomes study
OR: Odds Ratio
RR: Relative Risk
