# Supplementary material for: The index lift in data mining has a close relationship with the association measure relative risk in epidemiological studies
Source: BMC Med Inform Decis Mak. 2019 Jun 17;19:112. doi: 10.1186/s12911-019-0838-4 (PMC6580490; doi:10.1186/s12911-019-0838-4)
Supplement: Supplementary file 1 — Appendix 1: Relative risk derivation. Appendix 2: Odds ratio derivation. Appendix 3: Relative risk versus lift. Appendix 4: Trend of relative risk-lift ratio by association strength. Appendix 5: Trend of relative risk-lift ratio by exposure prevalence. Appendix 6: Theoretical relationship of lift-relative risk and lift-odds ratio for selected lift and exposure prevalence combinations. Appendix 7: Obtaining relative risk and odds ratio from the output of the Kingfisher and R arules packages. Appendix 8: R code for obtaining relative risk and odds ratio from lift, support, and confidence. Appendix 9: SAS code for obtaining relative risk and odds ratio from lift, support and confidence. (DOCX 63 kb) [file 12911_2019_838_MOESM1_ESM.docx]

**The Index Lift in Data Mining has a Close Relationship with the Association Measure Relative Risk in Epidemiological Studies - Appendices**

Appendix 1: Relative Risk Derivation

Given P(O), P(E), and lift, the following is an expression for the relative risk (RR):

$$RR≝\frac{P\left( O | E \right)}{P\left( O \right|\overline{E})}$$

$$= \frac{{P\left( OE \right)}/{P\left( E \right)}}{{P\left( O\overline{E} \right)}/{P\left( \overline{E} \right)}}$$

$$=\frac{{P\left( O \right)P\left( E \right)lift}/{P\left( E \right)}}{\left( P\left( O \right)-P\left( OE \right) \right)/\left( 1-P\left( E \right) \right)}$$

$$= \frac{P\left( O \right)lift}{\left( P\left( O \right)-P\left( O \right)P\left( E \right)lift \right)/\left( 1-P\left( E \right) \right)}$$

$$RR=\frac{\left( 1-P\left( E \right) \right)lift}{1-P(E)lift} (1.1)$$

Appendix 2: Odds Ratio Derivation

Given P(O), P(E), and lift, the following are expressions for the odds ratio (OR):

$$OR≝\frac{{P\left( O | E \right)}/{P\left( \bar{O} | E \right)}}{{P\left( O | \overline{E} \right)}/{P\left( \bar{O} | \overline{E} \right)}}=\frac{{P\left( O | E \right)}/\left( 1-P\left( O | E \right) \right)}{{P\left( O | \overline{E} \right)}/\left( 1-P\left( O | \overline{E} \right) \right)}$$

$$= \frac{{\frac{P\left( OE \right)}{P\left( E \right)}}/\left( 1-\frac{P\left( OE \right)}{P\left( E \right)} \right)}{{\frac{P\left( O\overline{E} \right)}{P\left( \overline{E} \right)}}/\left( 1-\frac{P\left( O\overline{E} \right)}{P\left( \overline{E} \right)} \right)}$$

$$= \frac{{\frac{P\left( O \right)P\left( E \right)lift}{P\left( E \right)}}/\left( 1-\frac{P\left( O \right)P\left( E \right)lift}{P\left( E \right)} \right)}{{\frac{P\left( O \right)-P\left( OE \right)}{P\left( \overline{E} \right)}}/\left( 1-\frac{P\left( O \right)-P\left( OE \right)}{P\left( \overline{E} \right)} \right)}$$

$$= \frac{{P\left( O \right)lift}/\left( 1-P\left( O \right)lift \right)}{{\frac{P\left( O \right)-P\left( O \right)P\left( E \right)lift}{P\left( \overline{E} \right)}}/{\frac{P\left( \bar{E} \right)-P\left( O \right)+P\left( O \right)P\left( E \right)lift}{P\left( \overline{E} \right)}}}$$

$$OR= \frac{lift\left( P\left( \bar{E} \right)-P\left( O \right)+P\left( O \right)P\left( E \right)lift \right)}{\left( 1-P\left( E \right)lift \right)\left( 1-P\left( O \right)lift \right)}$$

Substitute $P\left( \bar{E} \right) \mathrm{by} 1-P\left( E \right)$:

$$OR= \frac{lift\left( 1-P\left( E \right)-P\left( O \right)+P\left( O \right)P\left( E \right)lift \right)}{\left( 1-P\left( E \right)lift \right)\left( 1-P\left( O \right)lift \right)}$$

$$OR= \frac{\frac{\left( 1- P\left( E \right) \right)lift}{1-P\left( E \right)lift}-P\left( O \right)lift}{1-P\left( O \right)lift} (2.1)$$

Recall: $\frac{\left( 1- P\left( E \right) \right)lift}{1-P\left( E \right)lift}=RR$

$$OR= \frac{RR-P\left( O \right)lift}{1-P\left( O \right)lift}$$

As $lift≝ \frac{P\left( O | E \right)}{P\left( O \right)}=\frac{confidence}{P(O)}$, we have $P\left( O \right)lift=confidence$, which leads to:

$$OR= \frac{RR-confidence}{1-confidence} \left( 2.2 \right)$$

Appendix 3: Relative Risk versus Lift

$$From equation \left( 1.1 \right): RR=\frac{\left( 1-P\left( E \right) \right)lift}{1-P(E)lift}$$

By definition, we also have:

$$P\left( E \right)lift=P\left( E \right)\frac{P\left( OE \right)}{P\left( O \right)P\left( E \right)}=\frac{P\left( OE \right)}{P\left( O \right)}\leq1$$

Therefore, $\left( 1-P(E)lift \right)\geq0$, which means the denominator in equation (1.1) is non-negative.

When $lift>1$, we have $P\left( E \right)lift>P\left( E \right)\mathrm{and}\left( 1-P(E)lift \right)<1-P(E)$. Then,

$$RR=\frac{\left( 1-P\left( E \right) \right)lift}{\left( 1-P\left( E \right)lift \right)}>\frac{\left( 1-P\left( E \right) \right)lift}{\left( 1-P\left( E \right) \right)} =lift$$

Therefore, when $lift>1, RR>lift.$

Similarly, when $lift<1$, $P(E)lift<P(E) \mathrm{and} \left( 1-P\left( E \right)\mathrm{lift} \right)>1-P(E)$, and

$$RR=\frac{\left( 1-P\left( E \right) \right)lift}{\left( 1-P\left( E \right)lift \right)}<\frac{\left( 1-P\left( E \right) \right)lift}{\left( 1-P\left( E \right) \right)} =lift$$

Therefore, when $lift<1, RR<lift$

With a rare exposure, P(E) is approximately zero, and we obtain:

$$RR=\frac{\left( 1-P\left( E \right) \right)lift}{\left( 1-P\left( E \right)lift \right)} \approx\frac{\left( 1-0 \right)lift}{\left( 1-0*lift \right)} =\frac{lift}{1} =lift$$

Appendix 4: Trend of Relative Risk-Lift Ratio by Association Strength

$$From equation \left( 1.1 \right): RR= \frac{\left( 1-P\left( E \right) \right)lift}{\left( 1-P\left( E \right)lift \right)}$$

$$\Phi=\frac{RR}{lift}= \frac{\left( 1-P\left( E \right) \right)lift}{\left( 1-P\left( E \right)lift \right)lift}= \frac{\left( 1-P\left( E \right) \right)}{\left( 1-P\left( E \right)lift \right)}$$

Set $lift$ = X, P(E) = B:

$$\Phi=\frac{\left( 1-B \right)}{\left( 1-BX \right)}$$

To assess the trend of relative risk-lift ratio by association strength, take derivative of $\Phi$ by $lift$:

$$\frac{d\Phi}{dX}=\frac{\left( 1-B \right)^{'}\left( 1-BX \right)-\left( 1-B \right)\left( 1-BX \right)^{'}}{\left( 1-BX \right)^{2}}$$

$$=\frac{B\left( 1-B \right)}{\left( 1-BX \right)^{2}}$$

As B = P(E), the exposure prevalence, 0 ≤ B ≤ 1, B(1-B) is always positive, and $\frac{d\Phi}{dX}$ is then always positive. Therefore, the ratio between relative risk and lift always increases. With lift $>1$, when lift increases, the ratio between relative risk and lift increases. This property implies that relative risk moves away from the null value at a faster rate than lift. With lift $<1$, when lift decreases, the ratio between relative risk and lift decreases, which also implies that relative risk moves away from the null value at a faster rate than lift. In summary, on both sides of the null value of one, relative risk always moves away at a higher rate than lift.

Appendix 5: Trend of Relative Risk-Lift Ratio by Exposure Prevalence

To assess the trend of relative risk-lift ratio by the exposure prevalence, take the derivative of $\Phi$ by P(E):

$$\frac{d\Phi}{dB}=\frac{\left( 1-B \right)^{'}\left( 1-BX \right)-\left( 1-B \right)\left( 1-BX \right)^{'}}{\left( 1-BX \right)^{2}}$$

$$=\frac{X-1}{\left( 1-BX \right)^{2}}$$

When lift (denoted as X) is greater than one, the derivative is positive, and the relative risk-lift ratio increases. Alternatively, when lift is less than one, the derivative is negative, and the relative risk-lift ratio decreases. With lift less than one, smaller lift and relative risk values imply a stronger negative correlation, and the decreased relative risk-lift ratio has the same interpretation as the increased ratio with lift greater than one. In summary, when the exposure prevalence increases, the relative difference between relative risk and lift always increases.

Appendix 6: Theoretical relationship of lift-relative risk and lift-odds ratio for selected lift and exposure prevalence combinations.

| lift |  | Exposure Prevalence | | | | | | | | | | |
| --- | --- | --- | --- | --- | --- | --- | --- | --- | --- | --- | --- | --- |
|  |  | 0.01 | 0.05 | 0.1 | 0.2 | 0.3 | 0.4 | 0.5 | 0.6 | 0.7 | 0.8 | 0.9 |
| 1.0 | RR (RR/lift) | 1.0 (1.0) | 1.0 (1.0) | 1.0 (1.0) | 1.0 (1.0) | 1.0 (1.0) | 1.0 (1.0) | 1.0 (1.0) | 1.0 (1.0) | 1.0 (1.0) | 1.0 (1.0) | 1.0 (1.0) |
|  | OR (OR/lift) | 1.0 (1.0) | 1.0 (1.0) | 1.0 (1.0) | 1.0 (1.0) | 1.0 (1.0) | 1.0 (1.0) | 1.0 (1.0) | 1.0 (1.0) | 1.0 (1.0) | 1.0 (1.0) | 1.0 (1.0) |
| 1.2 | RR (RR/lift) | 1.2 (1.0) | 1.2 (1.0) | 1.2 (1.0) | 1.3 (1.1) | 1.3 (1.1) | 1.4 (1.2) | 1.5 (1.3) | 1.7 (1.4) | 2.3 (1.9) | 6.0 (5.0) |  |
|  | OR (OR/lift) | 1.2 (1.0) | 1.2 (1.0) | 1.3 (1.0) | 1.3 (1.1) | 1.4 (1.1) | 1.4 (1.2) | 1.6 (1.3) | 1.8 (1.5) | 2.4 (2.0) | 6.7 (5.6) |  |
| 1.4 | RR (RR/lift) | 1.4 (1.0) | 1.4 (1.0) | 1.5 (1.0) | 1.6 (1.1) | 1.7 (1.2) | 1.9 (1.4) | 2.3 (1.7) | 3.5 (2.5) | 21.0 (15.0) |  |  |
|  | OR (OR/lift) | 1.5 (1.1) | 1.5 (1.1) | 1.5 (1.1) | 1.6 (1.2) | 1.8 (1.3) | 2.1 (1.5) | 2.6 (1.8) | 3.9 (2.8) | 24.3 (17.3) |  |  |
| 1.6 | RR (RR/lift) | 1.6 (1.0) | 1.7 (1.0) | 1.7 (1.1) | 1.9 (1.2) | 2.2 (1.3) | 2.7 (1.7) | 4.0 (2.5) | 16.0 (10.0) |  |  |  |
|  | OR (OR/lift) | 1.7 (1.1) | 1.8 (1.1) | 1.9 (1.2) | 2.1 (1.3) | 2.4 (1.5) | 3.0 (1.9) | 4.6 (2.9) | 18.9 (11.8) |  |  |  |
| 1.8 | RR (RR/lift) | 1.8 (1.0) | 1.9 (1.0) | 2.0 (1.1) | 2.3 (1.3) | 2.7 (1.5) | 3.9 (2.1) | 9.0 (5.0) |  |  |  |  |
|  | OR (OR/lift) | 2.0 (1.1) | 2.1 (1.2) | 2.2 (1.2) | 2.5 (1.4) | 3.1 (1.7) | 4.5 (2.5) | 10.8 (6.0) |  |  |  |  |
| 2.0 | RR (RR/lift) | 2.0 (1.0) | 2.1 (1.1) | 2.3 (1.1) | 2.7 (1.3) | 3.5 (1.8) | 6.0 (3.0) |  |  |  |  |  |
|  | OR (OR/lift) | 2.3 (1.1) | 2.4 (1.2) | 2.6 (1.3) | 3.1 (1.5) | 4.1 (2.1) | 7.3 (3.6) |  |  |  |  |  |
| 2.5 | RR (RR/lift) | 2.5 (1.0) | 2.7 (1.1) | 3.0 (1.2) | 4.0 (1.6) | 7.0 (2.8) |  |  |  |  |  |  |
|  | OR (OR/lift) | 3.1 (1.2) | 3.3 (1.3) | 3.7 (1.5) | 5.0 (2.0) | 9.0 (3.6) |  |  |  |  |  |  |
| 3.0 | RR (RR/lift) | 3.1 (1.0) | 3.4 (1.1) | 3.9 (1.3) | 6.0 (2.0) | 21.0 (7.0) |  |  |  |  |  |  |
|  | OR (OR/lift) | 3.9 (1.3) | 4.4 (1.5) | 5.1 (1.7) | 8.1 (2.7) | 29.6 (9.9) |  |  |  |  |  |  |
| 3.5 | RR (RR/lift) | 3.6 (1.0) | 4.0 (1.2) | 4.8 (1.4) | 9.3 (2.7) |  |  |  |  |  |  |  |
|  | OR (OR/lift) | 5.0 (1.4) | 5.7 (1.6) | 6.9 (2.0) | 13.8 (3.9) |  |  |  |  |  |  |  |
| 4.0 | RR (RR/lift) | 4.1 (1.0) | 4.8 (1.2) | 6.0 (1.5) | 16.0 (4.0) |  |  |  |  |  |  |  |
|  | OR (OR/lift) | 6.2 (1.6) | 7.3 (1.8) | 9.3 (2.3) | 26.0 (6.5) |  |  |  |  |  |  |  |
| 4.5 | RR (RR/lift) | 4.7 (1.0) | 5.5 (1.2) | 7.4 (1.6) | 36.0 (8.0) |  |  |  |  |  |  |  |
|  | OR (OR/lift) | 7.7 (1.7) | 9.2 (2.0) | 12.6 (2.8) | 64.6 (14.4) |  |  |  |  |  |  |  |
| 5.0 | RR (RR/lift) | 5.2 (1.0) | 6.3 (1.3) | 9.0 (1.8) |  |  |  |  |  |  |  |  |
|  | OR (OR/lift) | 9.4 (1.9) | 11.7 (2.3) | 17.0 (3.4) |  |  |  |  |  |  |  |  |

Blank cells correspond to impossible combinations of lift values and exposure prevalence. The outcome prevalence is set at 0.1 when the odds ratio is computed for this table.

Appendix 7: Obtaining Relative Risk and Odds Ratio from the Output of the Kingfisher and R arules Packages

Using the support, confidence, and lift values available from the output of the Kingfisher and R arules packages, relative risk and odds ratio were obtained as follows:

$$P\left( E \right)=\frac{P\left( OE \right)}{P\left( O | E \right)}=\frac{support}{confidence}$$

Relative risk and odds ratio were then obtained from P(E)and lift using equations (1.1) and (2.2).

$$RR=\frac{\left( 1-P\left( E \right) \right)lift}{1-P(E)lift}=\frac{\left( confidence-support \right)lift}{confidence-support*lift}$$

$$OR=\frac{RR-confidence}{1-confidence}$$

Appendix 8: R code for obtaining Relative Risk and Odds Ratio from lift, support, and confidence

lift_RR <- function(rules, support, confidence, lift){

k <- as.list(match.call())

c = which(names(rules) %in% c(k$support, k$confidence, k$lift))

support = eval(k$support, rules); confidence = eval(k$confidence, rules); lift = eval(k$lift, rules)

RR = (confidence - support)*lift / (confidence - support*lift)

OR = (RR - confidence) / (1 - confidence)

result <- data.frame(rules[-(c)], support, confidence, lift, RR, OR)

return(result)

}

*#Example*

#Create a set of rules with lift, support and confidence

rules = c("n-Hexane, Toluene -> SGA", "Cumene -> SGA", "Benzene, Ethylene -> SGA",

"Biphenyl -> SGA", "Methanol, n-Hexane -> SGA", "Ethylene, n-Hexane -> SGA")

support = c(0.0606, 0.0226, 0.0422, 0.0140, 0.0593, 0.0463)

cf = c(0.0952, 0.0961, 0.1000, 0.0914, 0.0959, 0.0994)

gamma = c(1.0688, 1.0795, 1.1233, 1.0265, 1.0771, 1.1163)

Kingfisher = data.frame(rules, support, cf, gamma)

#call the function

example = lift_RR(rules = Kingfisher, support = support, confidence = cf, lift = gamma)

head(example)

#save result as .csv

write.table(example, "example.csv", sep=",", row.name = F)

Appendix 9: SAS code for obtaining Relative Risk and Odds Ratio from lift, support and confidence

**%macro** lift_RR_OR(dataset, support, confidence, lift);

data &dataset.; set &dataset.;

RR = (&confidence. - &support.) * &lift. / (&confidence. - &support.*&lift.);

OR = (RR - &confidence.) / (**1** - &confidence.);

label &support. = "support P(OE)"

&confidence. = "confidence P(O|E)"

&lift. = "lift(O|E)"

RR = "Relative Risk (RR)"

OR = "Odds Ratio (OR)";

proc print data = &dataset.(obs = **10**) noobs label;

run;

title "Summary: Lift to RR and OR";

proc means data = &dataset. n nmiss min max; var &lift. RR OR &support. &confidence.; run;

**%mend**;

*Example using Kingfisher report;

**data** Kingfisher;

input rules $**1**-**26** support cf gamma;

cards;

n-Hexane, Toluene -> SGA 0.0606 0.0952 1.0688

Cumene -> SGA 0.0226 0.0961 1.0795

Benzene, Ethylene -> SGA 0.0422 0.1000 1.1233

Biphenyl -> SGA 0.0140 0.0914 1.0265

Methanol, n-Hexane -> SGA 0.0593 0.0959 1.0771

Ethylene, n-Hexane -> SGA 0.0463 0.0994 1.1163

;

**run**;

*Run the macro;

%***lift_RR_OR***(Kingfisher, support, cf, gamma);

**proc** **export** data = Kingfisher

outfile = "C:\Kingfisher.csv" dbms = csv label replace; putnames = yes;

**run**;
